# Rad5 and Ubc4 directly ubiquitinate PCNA at Lys164 *in vitro*

**DOI:** 10.1016/j.jbc.2025.108192

**Published:** 2025-01-16

**Authors:** Yixiong Hu, Kaiyang Liu, Xue Bai, Pu Chen, Kai Zhang, Song Xiang

**Affiliations:** Department of Biochemistry and Molecular Biology, Key Laboratory of Immune Microenvironment and Disease (Ministry of Education), The Province and Ministry Co-sponsored collaborative Innovation Center for Medical Epigenetics, Tianjin Key Laboratory of Medical Epigenetics, Tianjin Medical University, Tianjin, PR China

**Keywords:** ubiquitination, ubiquitin ligase, ubiquitin-conjugating enzyme, DNA damage response, PCNA, Rad5, Ubc4

## Abstract

Ubiquitination of the proliferating cell nuclear antigen (PCNA) by the budding yeast protein Rad5 have important functions in replication stress responses. Rad5 together with the Ubc13–Mms2 complex attaches Lys63-linked ubiquitin chain to a highly conserved Lys164 residue in PCNA. The reaction requires prior PCNA monoubiquitination by the Rad6–Rad18 complex and signals for error-free DNA damage tolerance responses. Cellular studies suggested that Rad5 also cooperates with Ubc4 to catalyze PCNA ubiquitination in response to Okazaki fragment ligation defects, but biochemical evidence of this reaction is lacking. Here, we reconstituted this reaction and studied its biochemical properties. We found that Rad5 and Ubc4 directly ubiquitinate PCNA and the reaction requires a coordination of Rad5’s HIRAN and RING domains. Most interestingly, we found that the reaction ubiquitinates PCNA at multiple sites among which Lys164 is a major ubiquitination site. These findings suggest that Rad5 may contribute to replication stress responses through a novel mechanism by directly ubiquitinating Lys164 in PCNA.

The budding yeast protein Rad5 plays several critical roles in replication stress responses. Rad5 is targeted to stressed replication forks, where it recruits translesion synthesis (TLS) DNA polymerases to bypass the template lesions (1, 2, 3) or catalyze replication fork reversal to stabilize and protect the stalled replication fork (4, 5, 6). One of its most important functions is exerted through its ubiquitin ligase activity toward the proliferating cell nuclear antigen (PCNA). During DNA replication, PCNA is loaded onto DNA and recruits DNA polymerases and other factors for replication. At stressed replication forks, Rad5 together with the ubiquitin-conjugating complex Ubc13-Mms2 attaches Lys63-linked ubiquitin chain to a highly conserved Lys164 residue in PCNA. This modification signals for the error free branch of the DNA damage tolerance (DDT) pathway, allowing the stressed-stalled replication to resume *via* template switching. The reaction requires prior PCNA monoubiquitination at the same site by the Rad6–Rad18 complex, which signals for a somewhat error-prone TLS DDT branch (7, 8, 9, 10). These DDT responses are not only elicited by DNA damages but also by other replication stresses including defects in Okazaki fragment maturation (11, 12). They are highly conserved in eukaryotes. In human cells, Rad5 orthologs HLTF and SHPRH catalyze similar PCNA ubiquitination reactions and are implicated in many diseases including several types of cancer (13, 14, 15, 16, 17, 18).

In addition to the canonical PCNA ubiquitination reaction described above, studies on DNA ligase defective budding yeast cells have suggested a noncanonical PCNA ubiquitination reaction by Rad5. This reaction is likely induced by defects in Okazaki fragment ligation and signals for DNA damage responses (19, 20). In the fission yeast, a similar reaction facilitates the Rad52-dependent chromosomal rearrangements (21). These previous studies suggested that this noncanonical PCNA ubiquitination reaction also requires Mms2 but differs from the canonical PCNA ubiquitination reaction in several important aspects. First, the ubiquitin-conjugating enzyme in the noncanonical PCNA ubiquitination reaction is Ubc4; second, the noncanonical PCNA ubiquitination reaction does not require prior PCNA mono-ubiquitination; third, the non-canonical PCNA ubiquitination reaction attaches ubiquitin molecules to the Lys107 residue in PCNA. Unlike Lys164, Lys107 is conserved in fungi and *Caenorhabditis elegans* but not in higher eukaryotes. Nevertheless, it was found that in human cells DNA ligase deficiency also induces PCNA ubiquitination, but the ubiquitination site and the enzymes catalyzing the reaction remain to be identified (19).

Biochemical studies have provided mechanistic insights into the canonical PCNA ubiquitination reaction by Rad5 and its human homologs (22, 23, 24, 25, 26, 27). It was found that the reaction most likely proceeds *via* chain extension, in which the RING domain in Rad5 or homologous proteins catalyzes the addition of ubiquitin molecules to an ubiquitin chain already attached to PCNA. Rad5’s HIRAN domain at the N-terminal region interacts with PCNA and coordinates with the catalytic RING domain in the reaction (26, 27). In contrast, biochemical evidence of the noncanonical PCNA ubiquitination reaction by Rad5 is lacking. PCNA and components that may participate in this reaction are highly conserved in fungal species. For instance, the *Kluyveromyces lactis* PCNA (KlPCNA), Rad5 (KlRad5), Ubc4 (KlUbc4), and Mms2 (KlMms2) share 70.5%, 42.2%, 93.2%, and 83.2% sequence identity with the homologous budding yeast proteins, respectively. We have recently characterized the structure and action mechanisms of KlRad5 (27). Here, using purified KlRad5 and related proteins, we reconstituted the non-canonical PCNA ubiquitination reaction and characterized its biochemical properties. Our data show that KlRad5 and KlUbc4 catalyze robust PCNA ubiquitination reaction and the reaction requires a coordination of Rad5’s HIRAN and RING domains. Surprisingly, we found that KlMms2 does not alter the overall KlPCNA ubiquitination by KlRad5 and KlUbc4, and the reaction ubiquitinates multiple sites in KlPCNA in which the highly conserved Lys164 is a major ubiquitination site.

## Results

### Rad5 and Ubc4 directly ubiquitinate free PCNA and PCNA loaded onto DNA

**Figure 1:**
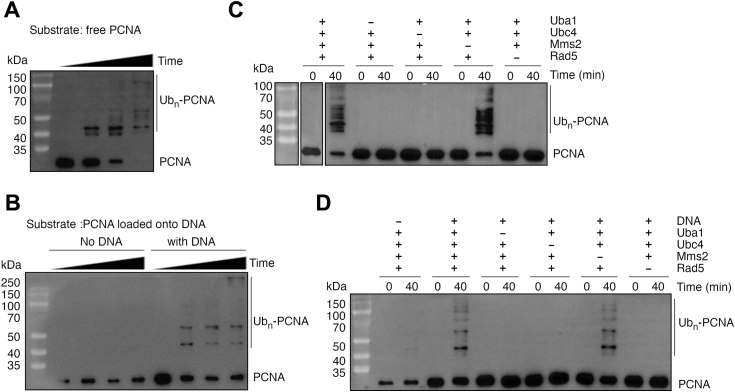
**Rad5 and Ubc4 directly ubiquitinate PCNA.***A* and *B*, ubiquitination reaction of free KlPCNA (*A*) and KlPCNA loaded onto DNA(*B*) by KlRad5 and KlUbc4. *C* and *D*, contribution of reaction components in the ubiquitination reaction of free KlPCNA (*C*) and KlPCNA loaded onto DNA (*D*). Western blot analyses of the reactions are presented. The reactions were allowed to proceed for 0, 5, 15, and 40 min before analysis, unless otherwise indicated. PCNA, proliferating cell nuclear antigen; KlPCNA, *Kluyveromyces lactis* PCNA.

To reconstitute the noncanonical PCNA ubiquitination reaction by Rad5 and Ubc4, we purified KlPCNA, KlRad5, KlUbc4, and KlMms2 (Fig. S1) and initiated the reaction by incubating them with the budding yeast ubiquitin-activating enzyme Uba1, ubiquitin, and ATP. Western blotting analysis for KlPCNA revealed that the reaction produced multiple modified PCNA species, whose molecular weights are consistent with KlPCNA attached with multiple ubiquitin molecules (Fig. 1*A*). With the increase of reaction time, higher molecular weight species emerge, the amount of the modified KlPCNA species increases, and the amount of unmodified KlPCNA decreases. These data suggests that KlRad5 and KlUbc4 can efficiently ubiquitinate free KlPCNA.

The trimeric PCNA ring is loaded onto DNA during DNA replication. PCNA loading onto DNA is required for its ubiquitination by the Rad6–Rad18 complex (28, 29). To test if KlRad5 and KlUbc4 can ubiquitinate PCNA loaded onto DNA, we purified DNA loaded KlPCNA following a previously published method (25). Briefly, we generated a circular DNA molecule containing multiple single- and double-stranded regions by annealing multiple primers to a circular ssDNA, loaded KlPCNA onto it with the budding yeast relipcation factor C (RFC) complex (Fig. S1), and purified the DNA loaded KlPCNA with strep-tactin resin that binds to one of the primers with a biotin tag. In a mock reaction without the DNA molecule, little PCNA was detected by Western blot (Fig. 1*B*), suggesting that the majority of the purified KlPCNA was loaded onto DNA. After incubating the DNA-loaded KlPCNA with KlRad5, KlUbc4, and related components, multiple modified KlPCNA species emerge, consistent with multiple ubiquitin molecules attached to KlPCNA (Fig. 1*B*). The data suggests that KlRad5 and KlUbc4 can also efficiently ubiquitinate KlPCNA loaded onto DNA.

### MmS2 does not alter the overall PCNA ubiquitination by Rad5 and Ubc4

Deleting Mms2 has been found to severely inhibit PCNA ubiquitination by Rad5 and Ubc4 in the budding yeast cell (19). To probe the role of Mms2 in the reaction, we removed KlMms2 from our reaction mixture. Surprisingly, we found that either free KlPCNA or the DNA-loaded KlPCNA can be efficiently ubiquitinated in the absence of KlMms2 (Fig. 1, *C* and *D*). In contrast, removing Uba1, KlRad5, or KlUbc4 completely abolished KlPCNA ubiquitination. An analysis of the reaction time course did not reveal any significant changes in reaction rate by removing KlMms2 in either the reaction with free KlPCNA or the reaction with KlPCNA loaded onto DNA (Fig. S2, *A* and *B*). Together, these data suggest that Mms2 does not alter the overall PCNA ubiquitination by Rad5 and Ubc4.

### Rad5’s RING and HIRAN domains coordinate for efficient PCNA ubiquitination

**Figure 2:**
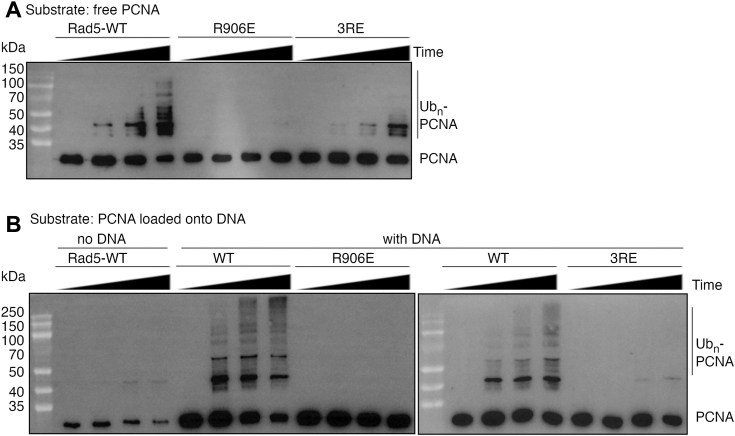
**Rad5’s HIRAN and RING domains coordinate for the noncanonical PCNA ubiquitination reaction.***A* and *B*, Western blot analysis of ubiquitination of free KlPCNA (*A*) and KlPCNA loaded onto DNA (*B*) by the WT and substituted KlRad5 and KlUbc4. The reactions were allowed to proceed for 0, 5, 15, and 40 min before analysis. PCNA, proliferating cell nuclear antigen; KlPCNA, *Kluyveromyces lactis* PCNA.

In the canonical PCNA ubiquitination reaction, Rad5’s RING domain catalyzes the reaction together with the Ubc13–Mms2 complex, whereas its HIRAN domain facilitates the reaction by interacting with PCNA (26, 27). The RING domain’s activity can be inhibited by the R906E substitution, which disrupts an important interaction with the Ubc13∼ubiquitin conjugate that induces its productive closed conformation. The mainly electrostatic HIRAN–PCNA interaction can be inhibited by the charge-reversal R190E/R228E/R240E (3RE) substitution in HIRAN domain’s PCNA-binding site (27). To test if the HIRAN-RING coordination also takes place in the noncanonical PCNA ubiquitination reaction, we measure activities of R906E- and 3RE-substituted KlRad5 variants. We found that in both reactions with free PCNA and PCNA loaded onto DNA, the R906E substitution eliminated KlRad5’s activity, whereas the 3RE substitution severely inhibited it (Fig. 2, *A* and *B*). These data suggest that the HIRAN-RING coordination is also required for efficient PCNA ubiquitination by Rad5 and Ubc4.

### Rad5 and Ubc4 ubiquitinate multiple PCNA sites

**Figure 3:**
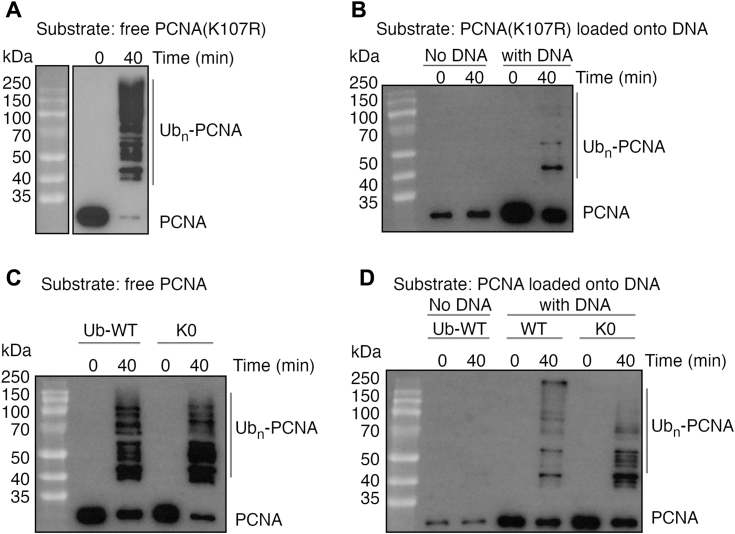
**Rad5 and Ubc4 ubiquitinate multiple PCNA sites.***A* and *B*, ubiquitination of free (*A*) and DNA loaded (*B*) K107R-substituted KlPCNA by KlRad5 and KlUbc4. *C* and *D*, ubiquitination of free (*C*) and DNA loaded (*D*) KlPCNA by KlRad5 and KlUbc4 with the K0 ubiquitin. Reactions with the WT ubiquitin are included for comparison. PCNA, proliferating cell nuclear antigen; KlPCNA, *Kluyveromyces lactis* PCNA.

Previous studies on budding yeast indicated that PCNA ubiquitination by Rad5 and Ubc4 was abolished by the K107R substitution in PCNA, suggesting that Lys107 is the ubiquitination site (19). Lys107 is highly conserved in fungal PCNA. Surprisingly, we found that KlRad5 and KlUbc4 can efficiently ubiquitinate the K107R-substituted KlPCNA (Fig. 3, *A* and *B*), suggesting that ubiquitination sites other than Lys107 exist in KlPCNA. Both ubiquitin chain attachment to KlPCNA and ubiquitination at multiple KlPCNA sites contribute to the multiply ubiquitinated KlPCNA products observed in our reaction. Substituting all lysine residues in ubiquitin to arginine (K0 ubiquitin) can eliminate ubiquitin chain formation, in which ubiquitin molecules are linked through its C-terminal glycine and a lysine residue side chain. To test if a single or multiple KlPCNA sites are ubiquitinated in the reaction, we analyzed reactions with the K0 ubiquitin. These reactions also produced multiply ubiquitinated KlPCNA species with either free KlPCNA or KlPCNA loaded onto DNA as the substrate (Fig. 3, *C* and *D*). The data suggest that KlRad5 and KlUbc4 ubiquitinate multiple KlPCNA sites. The number of bands for the ubiquitinated KlPCNA species suggests that 10 or more sites in KlPCNA could be ubiquitinated by KlRad5 and KlUbc4.

### Identification of PCNA ubiquitination sites by Rad5 and Ubc4

To identify the ubiquitination sites, we digested the reaction products and analyzed the resulting peptides by liquid chromatography (LC)-tandem mass spectrometry (MS/MS). The ubiquitination modification of a peptide was revealed by a mass increase of 114 Da, corresponding to the attachment of the ubiquitin C-terminal Gly-Gly motif. The ubiquitination sites were subsequently determined by analyzing the MS/MS fragment of the peptide. The analysis identified six ubiquitination sites in free KlPCNA, namely Lys33, Lys132, Lys164, Lys201 Lys210, and Lys217 (Table 1, Fig. S4, *A*–*F*, and Supplementary file 1). The peptide spectrum match (PSM) values associated with related peptides suggested that peptides containing ubiquitinated Lys33, Lys164, and Lys201 are significantly more abundant than peptides containing other ubiquitination sites (Table 1 and Supplementary file 1). The number of peptides detected by mass spectrometry (MS) for reactions with KlPCNA loaded onto DNA was significantly less (Supplementary file 1), consistent with the lower amount of KlPCNA we were able to recover from this reaction. Four ubiquitination sites were identified on DNA loaded KlPCNA, namely Lys33, Lys164, Lys201, and Lys210. The highest PSM values are associated with sites Lys164 and Lys201 (Table 1, Fig. S5, *A*–*D*, and Supplementary file 1).

**Table 1:** Summary of KlPCNA ubiquitination sites identified by mass spectrometry

Experiment round #	Reaction components			Ubiquitination sites identified on:	
	PCNA	Ub	Mms2	Free KlPCNA	KlPCNA loaded onto DNA
Round 1	WT	WT	-	33 (18), 132 (2), 164 (25), 201 (21), 210 (4), 217 (1)	33 (1), 164 (3), 201 (5), 210 (2)
	WT	WT	+	33 (25), 164 (26), 201 (21), 210 (4), 217 (1)	201 (1)
Round 2	6KR	WT	-	146 (1)	146 (3), 183 (2)
	6KR	K0	-	77 (3), 146 (2)	146 (2)
Round 3	9KR	K0	-	13 (6)	13 (1)

The PSM values associated with ubiquitinated peptides containing each site are enclosed in brackets.

−, absent; +, present; KlPCNA, *Kluyveromyces lactis* PCNA; PCNA, proliferating cell nuclear antigen; PSM, peptide spectrum match; Ub, ubiquitin.

The above MS experiments analyzed reactions without KlMms2. Although our enzymatic experiments indicated that KlMms2 does not alter the overall KlPCNA ubiquitination by KlRad5 and KlUbc4, these experiments could not reveal whether KlMms2 alter the preference of the ubiquitination sites. To test this possibility, we analyzed reactions with KlMms2 with MS. Compared to the reaction without KlMms2, identical ubiquitination sites were identified in free KlPCNA except for Lys132, which is associated with a low PSM (Table 1, Fig. S6, *A*–*E*, and Supplementary file 1). Among the identified sites, Lys33, Lys164, and Lys201 are also associated with significantly higher PSM values. MS analysis on the reaction with KlPCNA loaded onto DNA and KlMms2 identified one ubiquitination site, Lys201, which is also the site associated with the highest PSM value in the reaction without KlMms2 (Table 1, Fig. S7, and Supplementary file 1). Identification of additional sites in this reaction may be hampered by limited sample amount.

**Figure 4:**
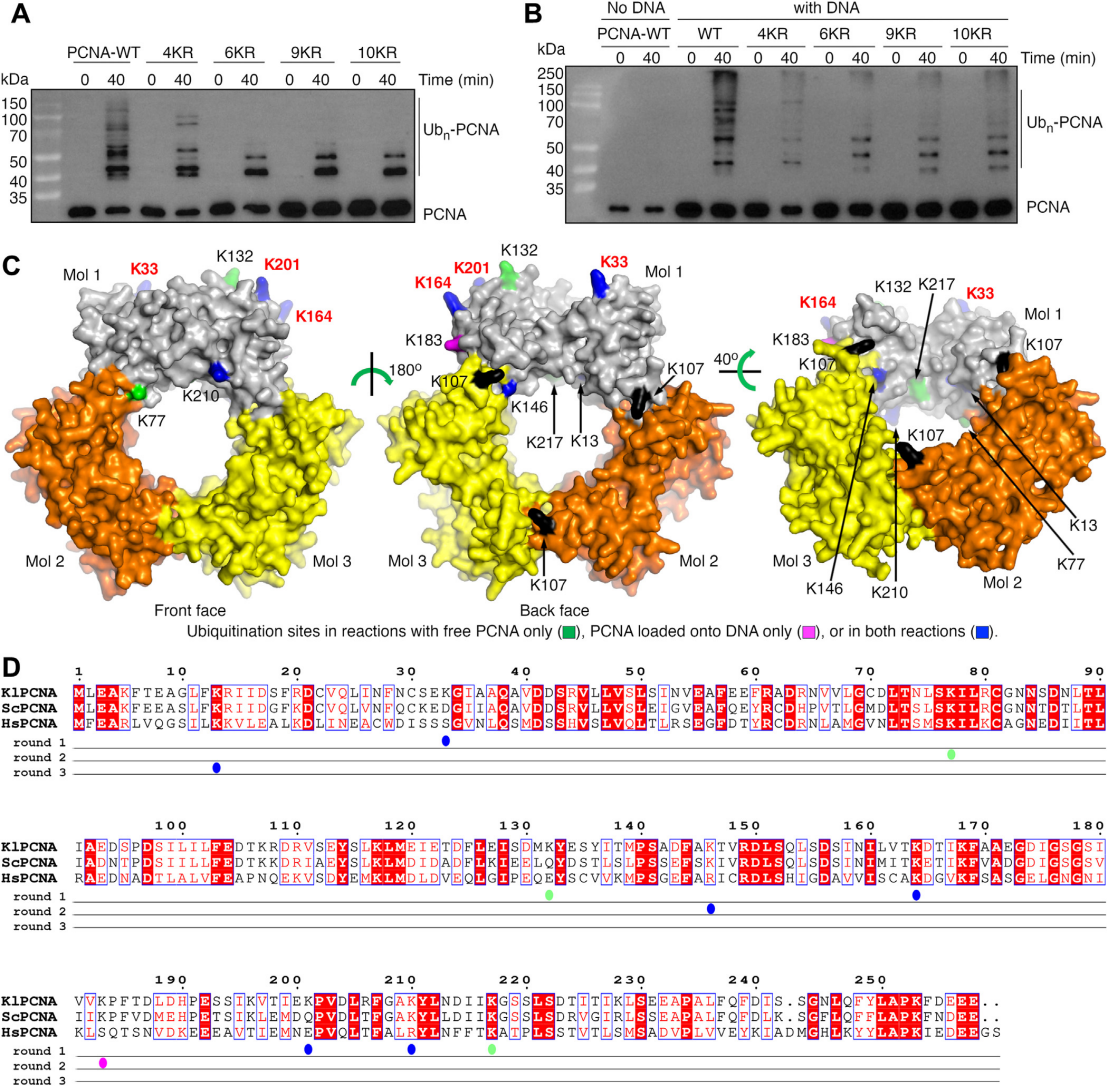
**Identification of PCNA ubiquitination sites by Rad5 and Ubc4.***A* and *B*, ubiquitination of the WT and substituted free KlPCNA (*A*) and DNA-loaded KlPCNA (*B*) by KlRad5 and KlUbc4. *C*, location of ubiquitination sites in KlPCNA. The KlPCNA structure was modeled based on the budding yeast PCNA structure (PDB: 4YHR). The three molecules in the KlPCNA trimer are colored in *gray*, *brown*, and *yellow*, respectively. For simplicity, only ubiquitination sites in molecule 1 are shown. Ubiquitination sites found only in free KlPCNA, only in DNA-loaded KlPCNA and in both free and DNA-loaded KlPCNA are colored in *green*, *magenta.* and *blue*, respectively. The potentially major ubiquitination sites, Lys33, Lys164, and Lys 201, are highlighted with *red labels*. Lys107 is highlighted in *black*. The front and back faces of PCNA are indicated. *D*, sequence alignment of PCNA. Sequences of KlPCNA, the budding yeast PCNA (ScPCNA) and the human PCNA (HsPCNA) are aligned. Sequence numbers are indicated above the sequences. *Dots* below the sequences indicate the ubiquitination sites identified in rounds 1 to 3 of mass spectrometry experiments. *Dots* are colored as the ubiquitination sites in *panel* (*C*). PCNA, proliferating cell nuclear antigen; KlPCNA, *Kluyveromyces lactis* PCNA.

Consistent with data obtained from the above MS analyses, incorporating arginine substitutions at Lys33, Lys164, Lys201, and Lys210 (4KR), or at Lys33, Lys132 Lys164, Lys201, Lys210, and Lys217 (6KR) in KlPCNA strongly inhibited its ubiquitination by KlRad5 and KlUbc4 (Figs. 4, *A* and *B*, and S3, *A* and *B*). However, KlPCNA ubiquitination was not eliminated, suggesting that additional ubiquitination sites exist. To identify these sites, we performed a second round of MS experiments on reactions with the 6KR-substituted KlPCNA. These experiments identified ubiquitination sites Lys146 for free KlPCNA, and Lys146 and Lys183 for KlPCNA loaded onto DNA (Table 1, Figs S8, S9, *A* and *B*, and Supplementary file 2). The ubiquitin chain formation reaction may inhibit ubiquitin attachment to KlPCNA by competing with it for ubiquitin. To promote ubiquitin attachment to KlPCNA, we replaced ubiquitin with the K0 ubiquitin in the reaction. MS experiments on these reactions identified ubiquitination sites Lys77 and Lys146 in free KlPCNA, and Lys146 in KlPCNA loaded onto DNA (Table 1, Fig. S10, *A* and *B*, S11, and Supplementary file 2).

Incorporating arginine substitutions at the three ubiquitination sites identified in the second round of MS experiments, Lys77, Lys146, and Lys183 into the 6KR-substituted KlPCNA (9KR) did not eliminate its ubiquitination by KlRad5 and KlUbc4 (Figs. 4, *A* and *B*, and S3, *A* and *B*), promoting us to perform a third round of MS experiments on reactions with the 9KR-substitued KlPCNA. These experiments identified one ubiquitination site, Lys13, in both free KlPCNA and KlPCNA loaded onto DNA (Table 1, Figs. S12, S13, and Supplementary file 3). Although incorporating the K13R substitution into the 9KR-substitued KlPCNA (10KR) did not eliminate its ubiquitination (Figs. 4, *A* and *B* and S3, *A* and *B*), MS experiments on reactions with the 10KR-substitued KlPCNA did not reveal any ubiquitination sites. It is likely that ubiquitination modification on additional KlPCNA sites are not substantial and below the detection threshold of MS.

Compared to ubiquitination sites identified in the first round of MS experiments, sites found in the second and third rounds are most likely minor sites, which only become substantially ubiquitinated when the major sites are eliminated. This is consistent with the relatively low PSM values associated with these sites and our enzymatic experiments. The latter show that the 4KR and 6KR substitutions drastically inhibited KlPCNA ubiquitination, whereas further inhibition by the 9KR and 10KR substitutions was not obvious (Figs. 4, *A* and *B* and S3, *A* and *B*). Among the ubiquitination sites identified by the first round of MS experiments, these associated with the highest PSM values are likely the major sites, since they are more frequently detected by MS. Together, the MS experiments suggests that KlRad5 and KlUbc4 ubiquitinate multiple sites in KlPCNA, in which Lys33, Lys164, and Lys201 are the major ubiquitination sites in free KlPCNA, whereas Lys164 and Lys201 are the major ubiquitination sites in DNA-loaded KlPCNA. Mapping the ubiquitination sites onto the predicted structure of KlPCNA indicates that the major sites are located at the back face of the trimeric PCNA ring, with Lys164 and Lys201 located close to each other (Figs. 4*C* and S14). These experiments also suggest that KlMms2 probably do not alter the preference of the ubiquitination sites we identified.

## Discussion

Here, we provide biochemical evidence that Rad5 together with Ubc4 directly ubiquitinates PCNA and the reaction requires a coordination of Rad5’s HIRAN and RING domains. Rad5 is well known for its ability to ubiquitinate PCNA that has been monoubiquitinated (7, 8, 9, 10). Its HIRAN domain has been found to be an interaction hub for DNA and PCNA and coordinate with other Rad5 domains in replication fork reversal and the canonical PCNA ubiquitination reaction by Rad5, Ubc13, and Mms2 (26, 27). Our findings extended the understanding of Rad5 and its HIRAN domain’s function.

Perhaps the biggest surprise it that we found that KlRad5 together with KlUbc4 ubiquitinates multiple KlPCNA sites. In addition to Lys164 and Lys107, previous studies have also indicated that Lys242 in the budding yeast PCNA can also be ubiquitinated and its ubiquitination plays a role in TLS (30). Our study suggests that yet additional PCNA sites can be ubiquitinated. Six of the ubiquitination sites we identified are conserved in the budding yeast, including Lys13, Lys77, Lys147, Lys164, Lys210, and Lys217 (Fig. 4*D*). The equivalent sites in the budding yeast PCNA may also be ubiquitinated by the budding yeast Rad5 and Ubc4. However, the sites we reported here were identified with *in vitro* experiments, further experiments are required to test whether they can be ubiquitinated *in vivo*. Notably, of the three sites we identified only in free KlPCNA, two (Lys77 and Lys217) are located close to or within the PCNA inner surface (Fig. 4*C*). DNA is expected to bind to the PCNA inner surface, which may block their ubiquitination in DNA-loaded PCNA in our experiments and inside the cell. During DNA replication, polymerases and additional factors are recruited to the PCNA front face, whereas its back face is exposed (Fig. S14) (31, 32, 33). The canonical ubiquitination site, Lys164, is in the back face and it was found that its ubiquitination is not inhibited by FEN1 binding to PCNA (30). In contrast, two of the sites we identified, Lys77 and Lys210, are in the front face (Fig. 4*C*). Their ubiquitination may be hindered by replication factors inside the cell.

Some important ubiquitination sites may have escaped detection by our experiments. For instance, although Lys107 is conserved in the budding yeast PCNA and KlPCNA (Fig. 4*D*), it was not identified as an ubiquitination site by our experiments. An analysis of the MS data indicated that peptides containing Lys107 were poorly detected. Such peptides were only detected in three of the 10 MS experiments (round 1 experiment with free KlPCNA and KlMms2, round 2 experiments with K0 ubiquitin) and were associated with low PSM values (Supplementary files 1–3). Therefore, a conclusion cannot be drawn from our data whether Lys107 in KlPCNA is ubiquitinated by KlRad5 and KlUbc4. Additional experiments are required to test this possibility.

All of the three major ubiquitination sites we identified are located in the back face of KlPCNA (Figs. 4*C* and S14). Among them, Lys33 and Lys201 are not conserved in the budding yeast PCNA (Fig. 4*D*). It remains to be seen whether their ubiquitination have unique functions in *K. lactis*. Most interestingly, the other major ubiquitination site we identified in both free PCNA and DNA-loaded PCNA, Lys164, is the highly conserved canonical PCNA ubiquitination site by the Rad6–Rad18 complex and Rad5. Lys164 ubiquitination plays critical roles in DDT responses (7, 8, 9, 10, 11, 12). Previous studies indicated that its polyubiquitination by Rad5 and the Ubc13–Mms2 complex requires prior monoubiquitination by the Rad6–Rad18 complex (7, 8, 9, 10). Our study suggests that Rad5 together with Ubc4 can directly ubiquitinate Lys164 in PCNA, bringing up the possibility that Rad5 may regulate replication stress responses through a novel mechanism. Given that Lys164 is highly conserved in eukaryotes, it is interesting to test whether this novel function of Rad5 is also possessed by HLTF and SHPRH.

Here, we found that KlMms2 did not alter the overall KlPCNA ubiquitination by KlRad5 and KlUbc4 or the preference of the ubiquitination sites we identified. Studies on budding yeast suggested that Mms2 is required for PCNA ubiquitination at Lys107 by Rad5 and Ubc4 (19). As we can hardly detect peptides containing Lys107 in our MS experiments, it remains to be seen whether KlMms2 plays a specific role in KlPCNA Lys107 ubiquitination by KlRad5 and KlUbc4. A well-studied function of Mms2 is linked to its complex formation with Ubc13, in which it positions the acceptor ubiquitin and brings its Lys63 side chain for isopeptide bond formation with the donor ubiquitin by Ubc13 (34, 35, 36). We were not able to detect a strong interaction between KlMms2 and KlUbc4. If Mms2 participates in the PCNA ubiquitination reaction by Rad5 and Ubc4, it may do so through a novel mechanism. Alternatively, Mms2 may be required for upstream events and does not directly contribute to the ubiquitination reaction. Further studies are required to understand the role of Mms2 in PCNA ubiquitination by Rad5 and Ubc4.

In addition to which PCNA sites are ubiquitinated, another important question to answer is whether these sites are monoubiquitinated or modified by ubiquitin chains (polyubiquitinated). If the latter is the case, it is also important to determine how ubiquitin molecules are linked in these ubiquitin chains. In our ubiquitination reactions with the K0 ubiquitin, less high molecular weight ubiquitinated KlPCNA species were produced (Fig. 3, *C* and *D*), suggesting that KlPCNA is polyubiquitinated at some of the ubiquitination sites. Our MS experiments identified several ubiquitinated ubiquitin residues, including Lys11, Lys33, Lys48, and Lys63 (Table 2, Figs. S15–S20, and Supplementary files 1–2), which probably mediate ubiquitin linkage in the ubiquitin chain. However, it is not clear whether these residues mediate the ubiquitin linkage in KlPCNA-attached ubiquitin chains or free ubiquitin chains that may also be produced in our reactions. The K0 substitution did not strongly inhibit the production of the multiply ubiquitinated KlPCNA product, especially in the reaction with free KlPCNA (Fig. 3, *C* and *D*). Therefore, it is likely that ubiquitination at multiple KlPCNA sites, rather than ubiquitin chain attachment to KlPCNA, contributed more to the observed multiply ubiquitinated PCNA product. It is difficult to determine the ubiquitin linkage in the PCNA-attached ubiquitin chains in such a reaction. Future studies are required to reveal the ubiquitin linkage in the PCNA-attached ubiquitin chain.

**Table 2:** Summary of ubiquitin ubiquitination sites identified by mass spectrometry

Experiment round #	Reaction components			Ubiquitination sites in reactions with:	
	PCNA	Ub	Mms2	Free KlPCNA	KlPCNA loaded onto DNA
Round 1	WT	WT	-	11 (33), 33 (2), 48 (11), 63 (22)	11 (6), 33 (2), 48 (5), 63 (14)
	WT	WT	+	11 (53), 33 (3), 48 (6), 63(27)	48 (2), 63 (4)
Round 2	6KR	WT	-	11 (4), 33 (7), 48 (24), 63 (8)	11 (3), 48 (4), 63 (9)
	6KR	K0	-	ND	ND
Round 3	9KR	K0	-	ND	ND

The PSM values associated with ubiquitinated peptides containing each site are enclosed in brackets.

−, absent; +, present; KlPCNA, *Kluyveromyces lactis* PCNA; ND, not detected; PCNA, proliferating cell nuclear antigen; PSM, peptide spectrum match; Ub, ubiquitin.

In summary, in this study we provided biochemical evidence that Rad5 and Ubc4 directly ubiquitinate PCNA at multiple sites in which Lys164 is a major site of ubiquitination, and the reaction requires a coordination of Rad5’s HIRAN and RING domains. We envision that our data could prompt future studies, especially on how the Rad5 and Ubc4 catalyzed PCNA Lys164 ubiquitination contribute to replication stress responses.

## Experimental procedures

### Proteins

KlRad5 (residues 163–1114) (27), ubiquitin (27) and the budding yeast Uba1 (residues 10–1024) (37) were expressed in *Escherichia coli* cells and purified following previously published methods. The expression and purification method of the budding yeast RFC complex was adopted from a previous study (38). Briefly, the RFC1 gene fragment encoding residues 295 to 798 was inserted into the vector pETDuet (Novagen), the RFC2 and RFC3 genes were inserted into the vector pCDFDuet (Novagen), and the RFC4 and RFC5 genes were inserted into the vector pRSFDuet (Novagen). The above three plasmids were cotransformed into BL21 (DE3) cells to express the RFC complex, which was purified by ion-exchange (Hitrap SP, GE Healthcare), nickel-nitrilotriacetic acid (Ni-NTA, Smart-Lifesciences) and size-exclusion (Superdex 200 increase 10/300, GE Healthcare) columns. The KlMms2 gene was inserted into the vector pET28A. KlMms2 with an N-terminal 6x histidine tag was expressed in *E. coli* BL21 Rosetta (DE3) cells and purified with Ni-NTA and size-exclusion (Superdex 200 10/300, GE Healthcare) columns. The *K. lactis* PCNA gene was inserted into vector pET28A, with an oligonucleotide encoding the hemagglutinin (HA) tag attached to its 5′ end. KlPCNA with N-terminal 6x histidine and HA tags were expressed in *E. coli* BL21 Rosetta (DE3) cells and purified by Ni-NTA, ion-exchange (Hitrap Q HP, GE Healthcare), and size-exclusion (Superdex 200 increase 10/300, GE Healthcare) columns. The *K. lactis* Ubc4 gene was inserted into the vector pET28A. KlUbc4 with an N-terminal 6x histidine tag was expressed in BL21 Rosetta (DE3) cells and purified with Ni-NTA and size-exclusion (Superdex 200 10/300) columns. Amino acid substitutions were generated by polymerase chain reactions following instructions of the QuikChange kit (Agilent Technologies) and verified by DNA sequencing. The expression and purification of the substituted proteins followed the same method for the WT proteins.

### Reconstitution of the PCNA ubiquitination reaction by Rad5 and Ubc4

The reaction mixture with free KlPCNA contains 40 mM Tris (pH7.5), 50 mM sodium chloride, 10 mM magnesium chloride, 1 mM ATP, 0.15 μM Uba1, 5 μM KlUbc4, 0.5 μM KlRad5, 30 μM ubiquitin, and 0.1 μM PCNA. The reactions were allowed to proceed at 30 °C for the indicated time and terminated by boiling in the SDS-PAGE loading buffer.

The circular DNA containing multiple single- and double-stranded regions were prepared following a previously published method (25). To purify the DNA loaded KlPCNA, 25 μl strep-tactin beads (Smart-Lifesciences) equilibrated in the reaction buffer (20 mM Tris (pH7.5), 50 mM sodium chloride, 10 mM magnesium chloride, 1 mM DTT, 0.2 mg/ml bovine serum albumin (Sango Biotech)) was incubated with 1 μg DNA at 4 °C for 1 h; after washing the beads with 1 ml binding buffer three times, the total volume was adjusted to 100 μl and 15 μM KlPCNA, 0.6 μM RFC, and 1 mM ATP were added; after another incubation at 30 °C for 10 min, unbound proteins were removed by washing the beads 6 times with the reaction buffer. The reaction mixture for DNA loaded KlPCNA was the same as the reaction mixture for the free KlPCNA, except that free PCNA was replaced by strep-tactin beads bound with purified DNA and KlPCNA (25 μl for every 100 μl of reaction), and 0.2 mg/ml of bovine serum albumin and 1 mM DTT was added to the reaction mixture. The reactions were allowed to proceed at 30 °C for the indicated time and terminated by boiling in SDS-PAGE loading buffer.

The reactions were analyzed by Western blotting with rabbit anti-HA (C29F4, Cell Signaling Technology, 1:3000 diluted) and horseradish peroxidase–conjugated goat anti-rabbit IgG (ZB-2301, ZSGB-BIO, 1:5000 diluted) antibodies.

### MS experiments

#### In-gel digestion

For MS analysis, the ubiquitination reaction products were fractionated by SDS-PAGE. The gel was stained by silver staining (Beyotime) and regions containing ubiquitinated PCNA species were sliced from the gel. The gel slices were incubated in an acidic buffer (acetic acid/ethanol/water, 1:5:4, (v/v/v) overnight, washed twice with water, and destained with a buffer containing 50 mM K_3_[Fe(CN)_6_] and 15 mM NaS_2_O_3_. After washing twice with water, the destained slices were cut into small pieces (1 mm), dehydrated in acetonitrile, and dried with a SpeedVac (Thermo Fisher Scientific). The pieces were incubated with 10 mM DTT at 56 °C for 1 h to break disulfide bonds in proteins, and subsequently with 55 mM iodoacetamide at room temperature for 45 min to protect the broken disulfate bonds. After washing twice with water, dehydration in acetonitrile and drying with a SpeedVac, the gel pieces were mixed with a buffer containing 200 ng trypsin (Promega) and 50 mM ammonium bicarbonate and incubated overnight at 37 °C. Digested peptides were extracted from the gel pieces with buffer 1 (50% acetonitrile, 45% water and 5% TFA), buffer 2 (75% acetonitrile, 25% water, and 0.1% TFA), and acetonitrile. The peptide extracts were pooled, dried with a SpeedVac, and desalted with a μ-C18 Ziptip (MilliporeSigma).

#### LC-MS/MS

The desalted sample was injected into a C18 column (75 μm inner diameter × 25 cm, 3 μm C18) on a Nano-LC system (EASY-nLC 1200, Thermo Fisher Scientific) with a flow rate of 300 nl/min. The column was equilibrated with buffer A (0.1% formic acid) and peptides were eluted from the column with the following gradient of buffer B (0.1% formic acid in 80% acetonitrile): 5% to 6% in 1 min, 6% to 25% in 32 min, 25% to 38% in 8 min, 38% to 100% in 2 min, and hold at 100% for 6 min. The eluent was electrosprayed directly into an Orbitrap Eclipse mass spectrometer (Thermo Fisher Scientific). The source was operated at 2.2 kV. The mass spectrometric analysis was carried out in a data-dependent mode. For the MS1 survey scan, automatic gain control target is 4e5 and the resolution is 60,000. The MS2 spectra were acquired with a resolution of 30,000.

#### Data processing

MS/MS data were analyzed with Proteome Discoverer 3.0 (Thermo Fisher Scientific) with an overall false discovery rate for peptides of less than 1%. Peptide sequences were searched using trypsin specificity allowing a maximum of two missed cleavages. Carbamidomethylation on cysteine was specified as fixed modification. Oxidation of methionine, acetylation on the peptide N-terminal and ubiquitin on lysine were specified as variable modifications. Mass tolerances for precursor ions were set at ±10 ppm for precursor ions and ± 0.02 Da for MS/MS.

## Data availability

Original scans of the gels and blots and mass spectrometry data are available upon request.

## Supporting information

This article contains the following supporting information files.

Supporting information file 1 Summary of ubiquitination sites identified by the first round of mass spectrometry experiments. Sheets 1 to 4 list peptides identified in reactions with free KlPCNA and without KlMms2, with DNA-loaded KlPCNA and without KlMms2, with free KlPCNA and KlMms2, and with DNA-loaded KlPCNA and KlMms2, respectively. Ubiquitinated peptides highlighted in green are accompanied with high-confidence MS/MS spectra and are included in Tables 1 and 2. Ubiquitinated peptides highlighted in brown or not highlighted are accompanied with low-confidence MS/MS spectra or no MS/MS spectrum, respectively.

Supporting information file 2 Summary of ubiquitination sites identified by the second round of mass spectrometry experiments. Sheets 1 to 4 list peptides identified in reactions with free 6KR-substituted KlPCNA and the WT ubiquitin, with DNA-loaded 6KR-substituted KlPCNA and the WT ubiquitin, with free 6KR-substituted KlPCNA and the K0 ubiquitin, and with DNA-loaded 6KR-substituted KlPCNA and the K0 ubiquitin, respectively.

Supporting information file 3 Summary of ubiquitination sites identified by the third round of mass spectrometry experiments. Sheets 1 to 2 list peptides identified in reactions with free 9KR-substituted KlPCNA and K0 ubiquitin, with DNA-loaded 9KR-substituted KlPCNA and K0 ubiquitin, respectively.

Supporting information file 4Figs. S1–S20.

## Conflict of interest

The authors declare that they have no conflicts of interest with the contents of this article.

## Supporting information

Round 1

Round 2

Round 3
