# Supplementary material for: Rad5 and Ubc4 directly ubiquitinate PCNA at Lys164 in vitro
Source: J Biol Chem. 2025 Jan 16;301(3):108192. doi: 10.1016/j.jbc.2025.108192 (PMC11871451; doi:10.1016/j.jbc.2025.108192)
Supplement: Supporting information [file mmc4.pdf]

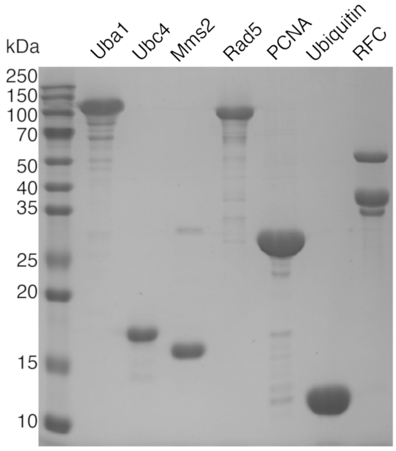

Figure S1 Proteins and the RFC complex used in this study. SDS PAGE analysis of the purified proteins and the RFC complex is presented.

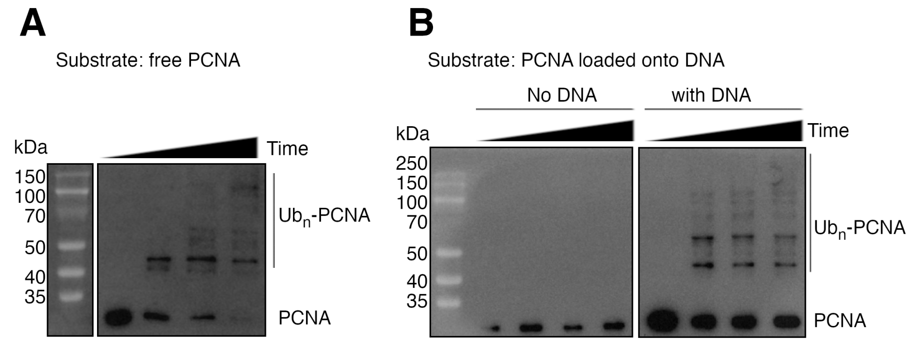

Figure S2 Mms2 is dispensable for PCNA ubiquitination by Rad5 and Ubc4. (A) and (B) Ubiquitination of free (A) and DNA loaded (B) KlPCNA by KlRad5 and KlUbc4 in the absence of KlMms2. In panel (B), mock experiments without DNA are included for comparison. They are identical to these presented in Fig. 1B.

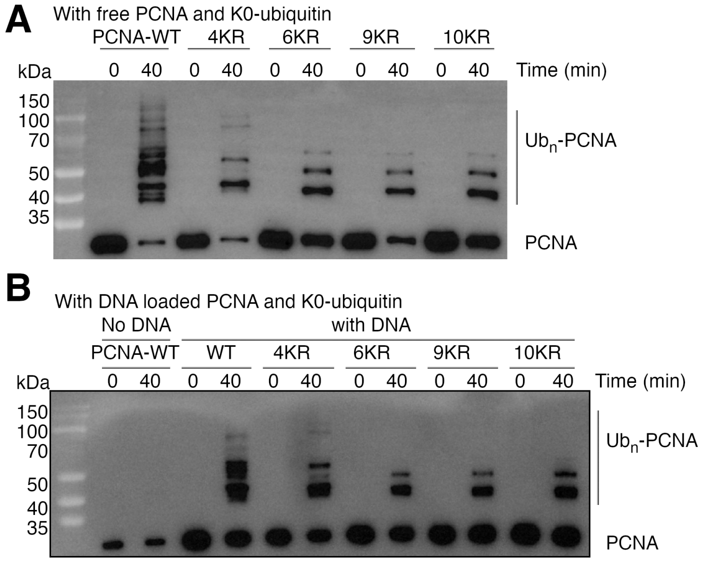

Figure S3 Ubiquitination of the wild type and substituted free KlPCNA (A) and DNA loaded PCNA (B) by KlRad5 and KlUbc4 with the K0 ubiquitin.

A

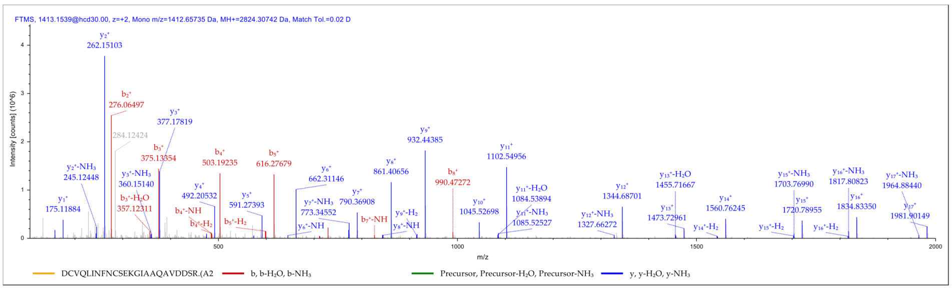

Ubiquitination site: Lys33
Sequence: DCVQLINFNCSEKGIAAQAVDDSR, C2-Carbamidomethyl (57.02146 Da), C10-Carbamidomethyl (57.02146 Da), K13-Ub (114.04293 Da)

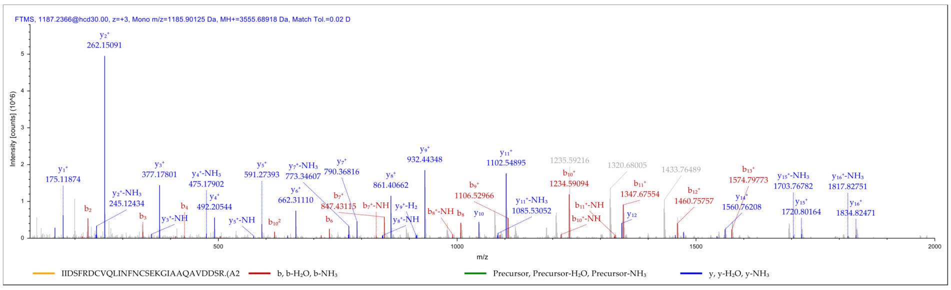

Ubiquitination site: Lys33
Sequence: IIDSFRDCVQLINFNCSEKGIAAQAVDDSR, C8-Carbamidomethyl (57.02146 Da), C16-Carbamidomethyl (57.02146 Da), K19-Ub (114.04293 Da)

B

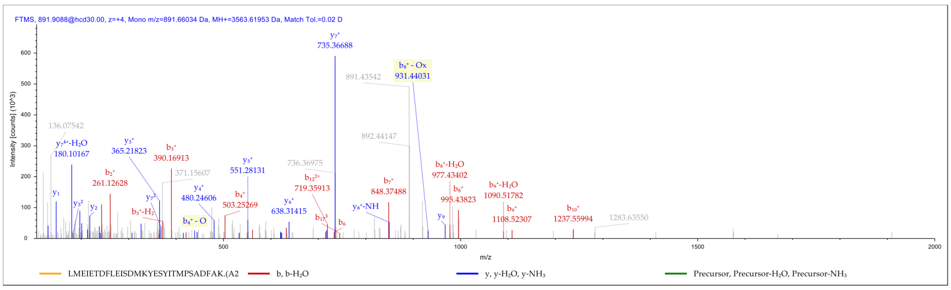

Ubiquitination site: Lys132
Sequence: LMEIETDFLEISDMKYESYITMPSADFAK, M2-Oxidation (15.99492 Da), M14-Oxidation (15.99492 Da), K15-Ub (114.04293 Da)

C

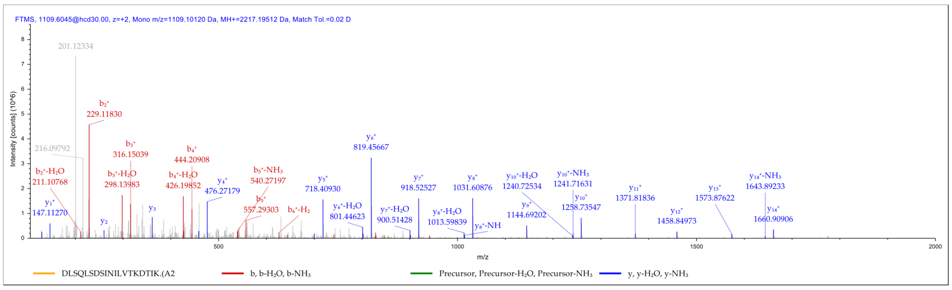

Ubiquitination site: Lys164
Sequence: DLSQLSDSINILVTKDTIK, K15-Ub (114.04293 Da)

Continues on the next page

Continued from the previous page

D

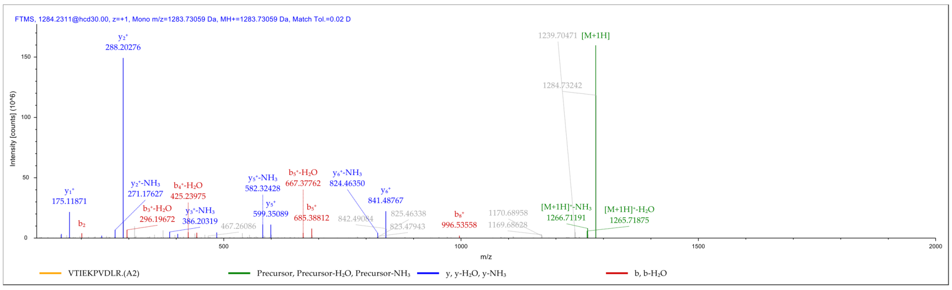

Ubiquitination site: Lys201
Sequence: VTIEKPVDLR, K5-Ub (114.04293 Da)

E

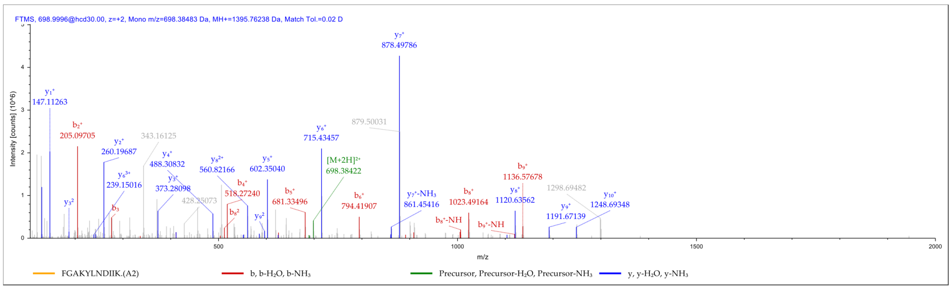

Ubiquitination site: Lys210
Sequence: FGAKYLNDIIK, K4-Ub (114.04293 Da)

F

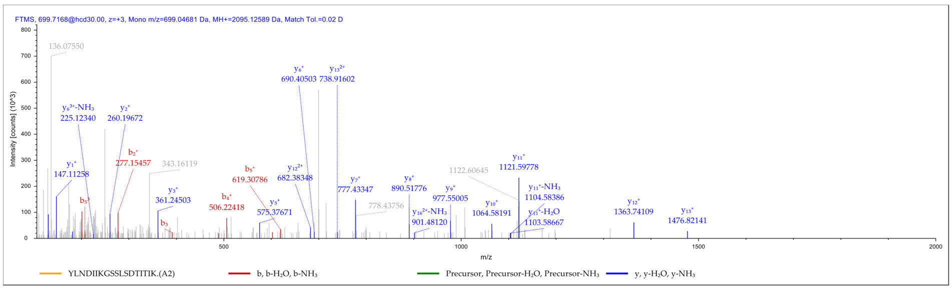

Ubiquitination site: Lys217
Sequence: YLNDIIKGSSLSDTITIK, K7-Ub (114.04293 Da)

Figure S4 MS/MS spectra of KlPCNA ubiquitination sites found in the reaction with free KlPCNA and without KlMms2.

A

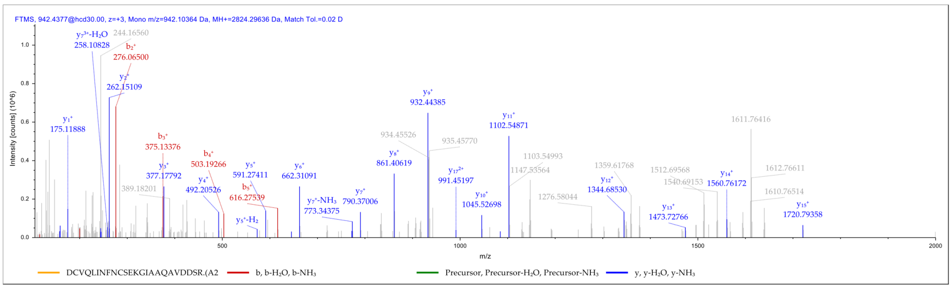

Ubiquitination site: Lys33
Sequence: DCVQLINFNCSEKGIAAQAVDDSR, C2-Carbamidomethyl (57.02146 Da), C10-Carbamidomethyl (57.02146 Da), K13-Ub (114.04293 Da)

B

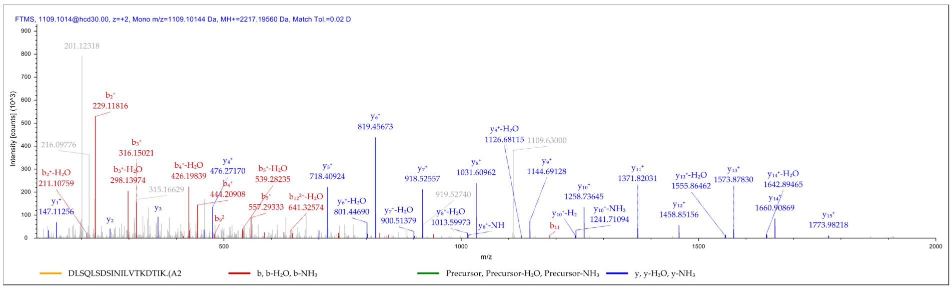

Ubiquitination site: Lys164
Sequence: DLSQLSDSINILVTKDTIK, K15-Ub (114.04293 Da)

C

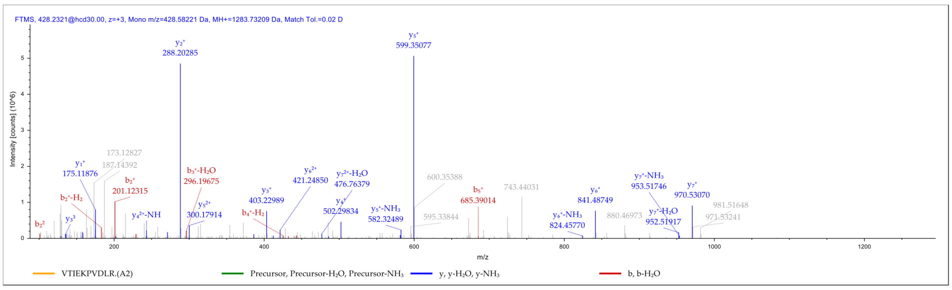

Ubiquitination site: Lys201
Sequence: VTIEKPVDLR, K5-Ub (114.04293 Da)

D

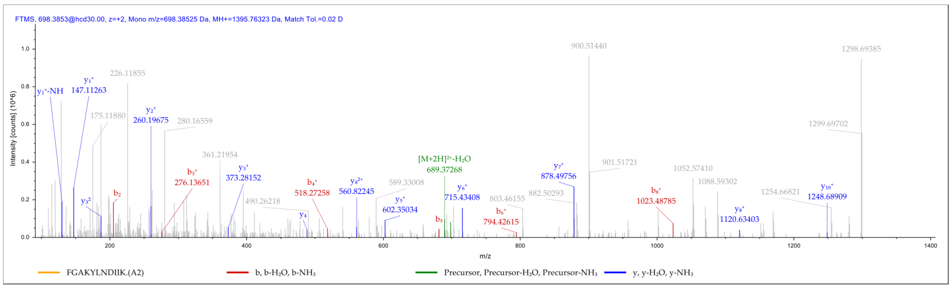

Ubiquitination site: Lys210
Sequence: FGAKYLNDIIK, K4-Ub (114.04293 Da)

Figure S5 MS/MS spectra of KlPCNA ubiquitination sites found in the reaction with DNA loaded KlPCNA and without KlMms2.

A

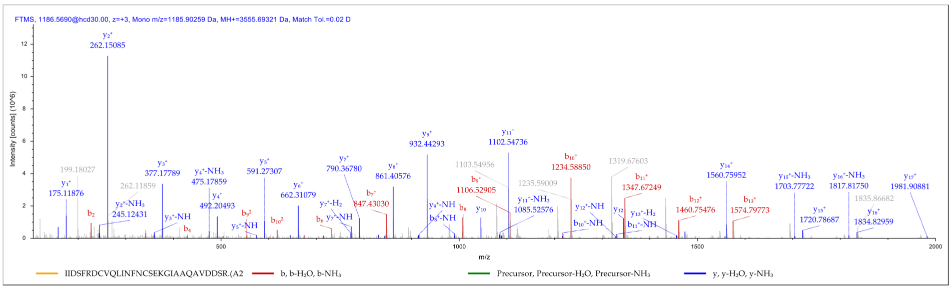

Ubiquitination site: Lys33
Sequence: IIDSFRDCVQLINFNCSEKGIAAQAVDDSR, C8-Carbamidomethyl (57.02146 Da), C16-Carbamidomethyl (57.02146 Da), K19-Ub (114.04293 Da)

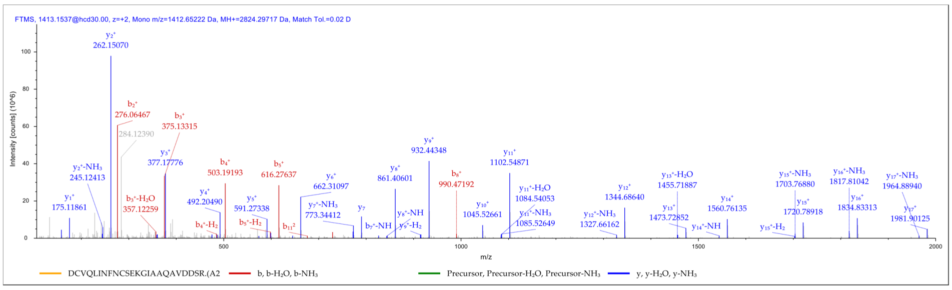

Ubiquitination site: Lys33
Sequence: DCVQLINFNCSEKGIAAQAVDDSR, C2-Carbamidomethyl (57.02146 Da), C10-Carbamidomethyl (57.02146 Da), K13-Ub (114.04293 Da)

B

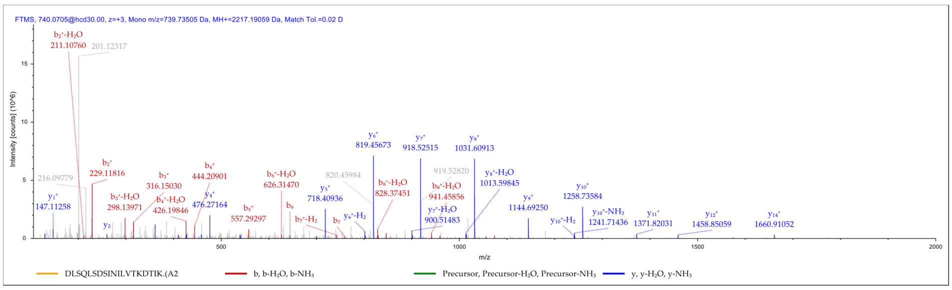

Ubiquitination site: Lys164
Sequence: DLSQLSDSINILVTKDTIK, K15-Ub (114.04293 Da)

C

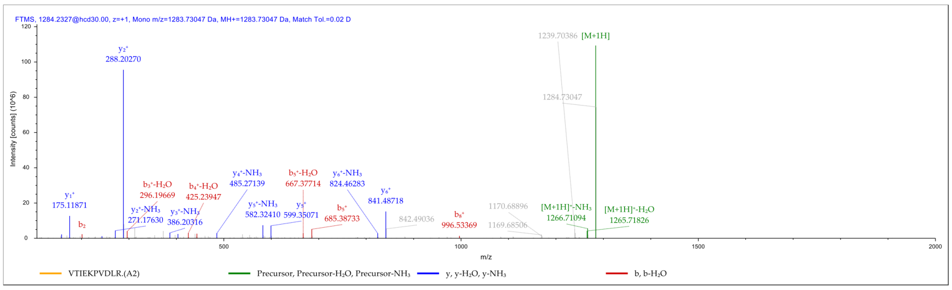

Ubiquitination site: Lys201
Sequence: VTIEKPVDLR, K5-Ub (114.04293 Da)

Continues on the next page

Continued from the previous page

D

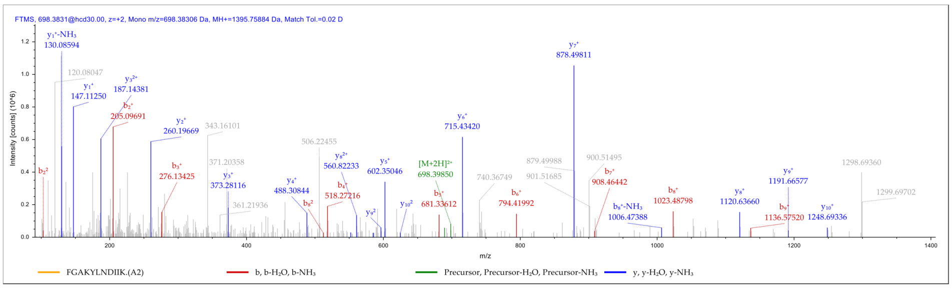

Ubiquitination site: Lys210
Sequence: FGAKYLNDIIK, K4-Ub (114.04293 Da)

E

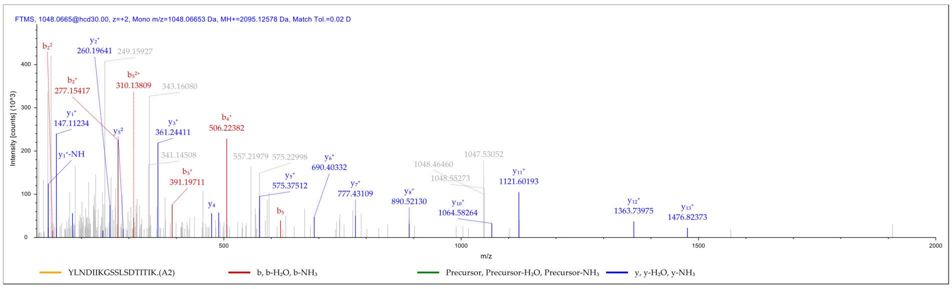

Ubiquitination site: Lys217
Sequence: YLNDIIKGSSLSDTITIK, K7-Ub (114.04293 Da)

Figure S6 MS/MS spectra of KlPCNA ubiquitination sites found in the reaction with free KlPCNA and KlMms2.

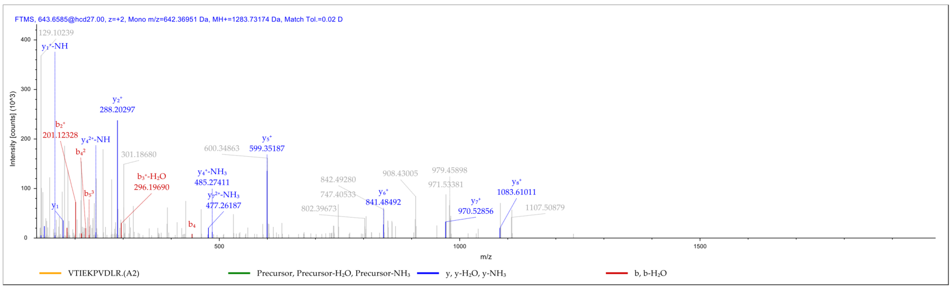

Ubiquitination site: Lys201
Sequence: VTIEKPVDLR, K5-Ub (114.04293 Da)

Figure S7 MS/MS spectrum of the KlPCNA ubiquitination site found in the reaction with DNA loaded KlPCNA and KlMms2.

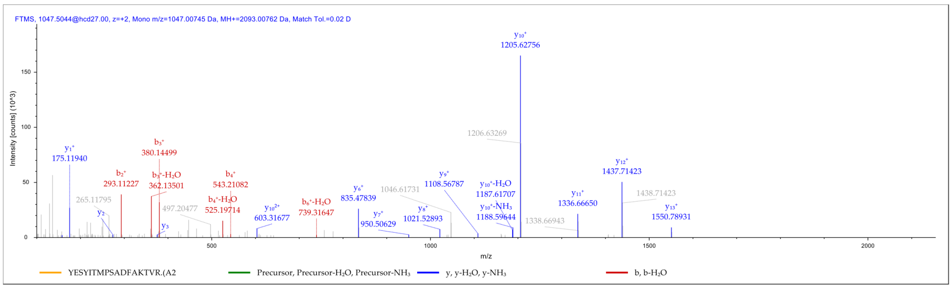

Ubiquitination site: Lys146
Sequence: YESYITMPSADFAKTVR, K14-Ub (114.04293 Da)

Figure S8 MS/MS spectrum of the KlPCNA ubiquitination site found in the reaction with free 6KR-substituted KlPCNA and the wild type ubiquitin.

A

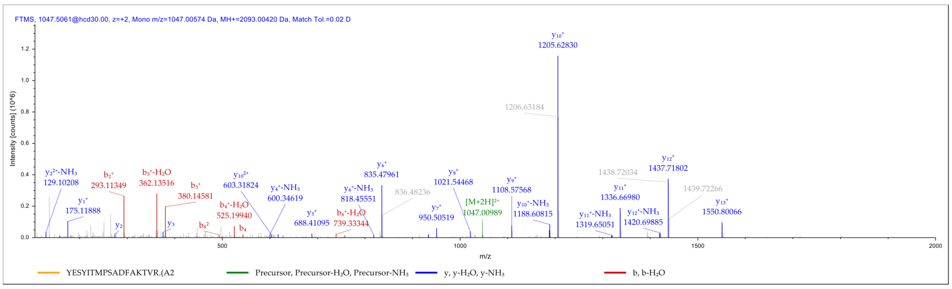

Ubiquitination site: Lys146
Sequence: YESYITMPSADFAKTVR, K14-Ub (114.04293 Da)

B

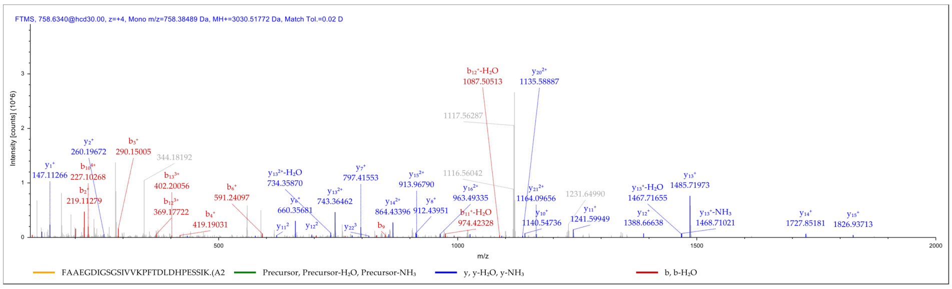

Ubiquitination site: Lys183
Sequence: FAAEGDIGSGSIVVKPFTDLDHPESSIK, K15-Ub (114.04293 Da)

Figure S9 MS/MS spectra of KlPCNA ubiquitination sites found in the reaction with DNA loaded 6KR-substituted KlPCNA and the wild type ubiquitin.

A

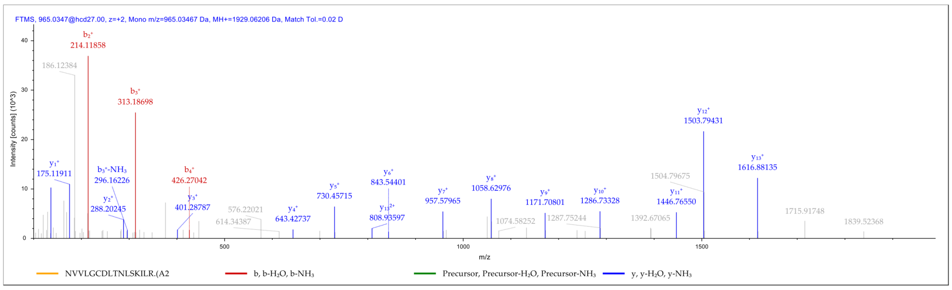

Ubiquitination site: Lys77
Sequence: NVVLGCDLTNLSKILR, C6-Carbamidomethyl (57.02146 Da), K13-Ub (114.04293 Da)

B

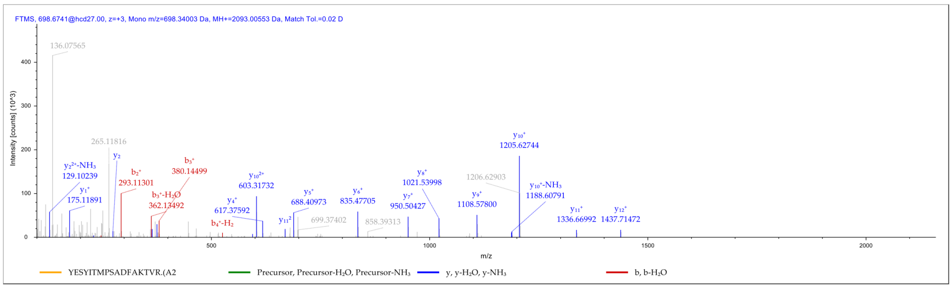

Ubiquitination site: Lys146
Sequence: YESYITMPSADFAKTVR, K14-Ub (114.04293 Da)

Figure S10 MS/MS spectra of KlPCNA ubiquitination sites found in the reaction with free 6KR-substituted KlPCNA and the K0 ubiquitin.

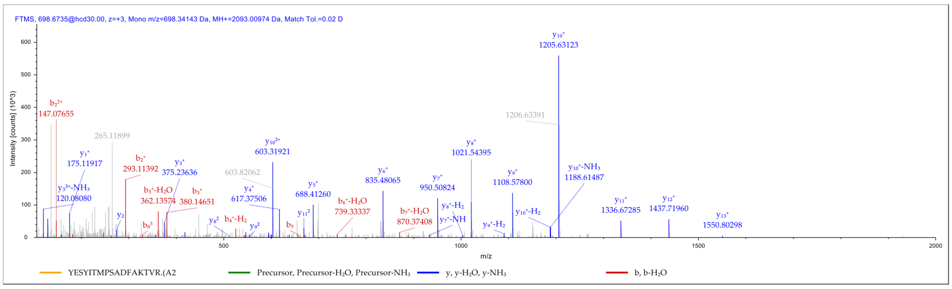

Ubiquitination site: Lys146
Sequence: YESYITMPSADFAKTVR, K14-Ub (114.04293 Da)

Figure S11 MS/MS spectrum of the KlPCNA ubiquitination site found in the reaction with DNA loaded 6KR-substituted KlPCNA and the K0 ubiquitin.

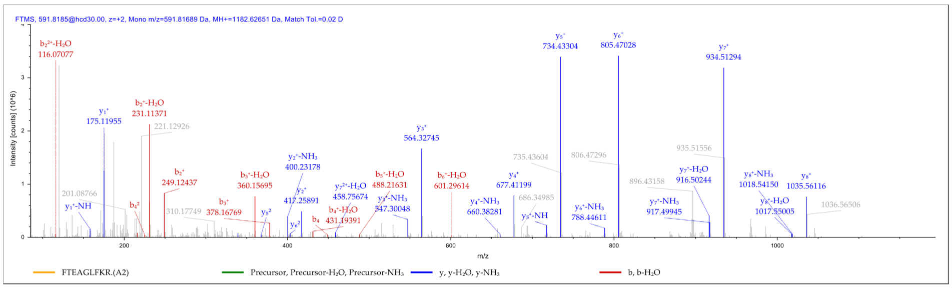

Ubiquitination site: Lys13
Sequence: FTEAGLFKR, K8-Ub (114.04293 Da)

Figure S12 MS/MS spectrum of the KlPCNA ubiquitination site found in the reaction with free 9KR-substituted KlPCNA and the K0 ubiquitin.

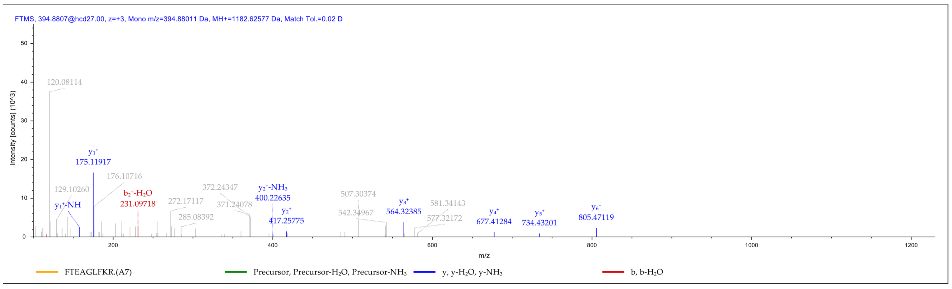

Ubiquitination site: Lys13
Sequence: FTEAGLFKR, K8-Ub (114.04293 Da)

Figure S13 MS/MS spectrum of the KlPCNA ubiquitination site found in the reaction with DNA loaded 9KR-substituted KlPCNA and the K0 ubiquitin.

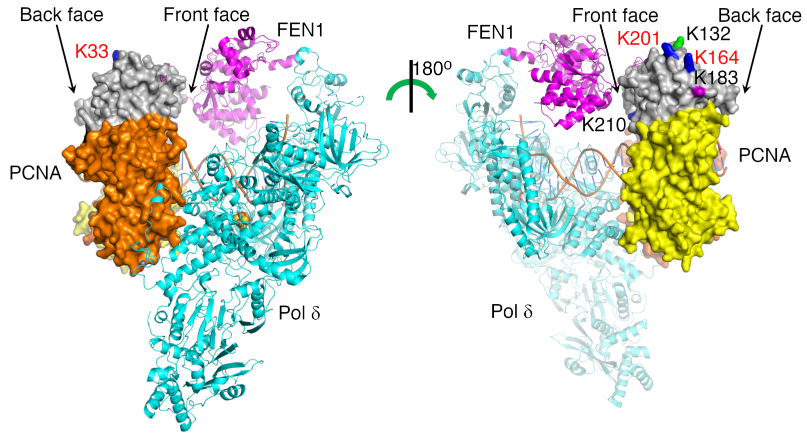

Figure S14 Front and back faces of PCNA. Strucure of the human PCNA in complex with DNA replication factors (Pol δ and FEN1, PDB 6TNZ) is presented. KlPCNA is superimposed on the human PCNA and presented as in Fig. 4C. Pol δ (cyan), FEN1 (magenta) and DNA (brown for backbone atoms) are presented in cartoon represntation. For clarity, the human PCNA is not shown.

A

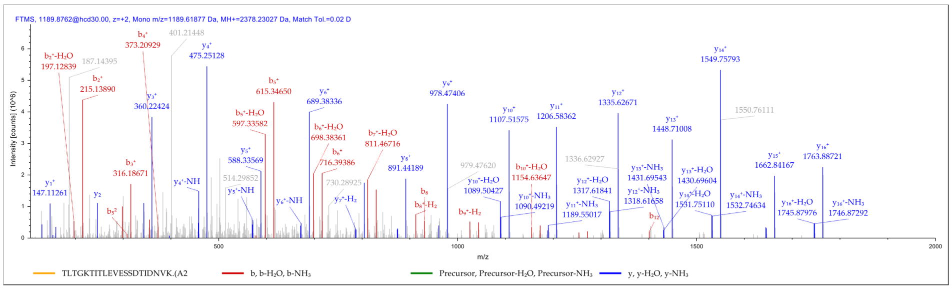

Ubiquitination site: Lys11
Sequence: TLTGKTITLEVESSDTIDNVK, K5-Ub (114.04293 Da)

B

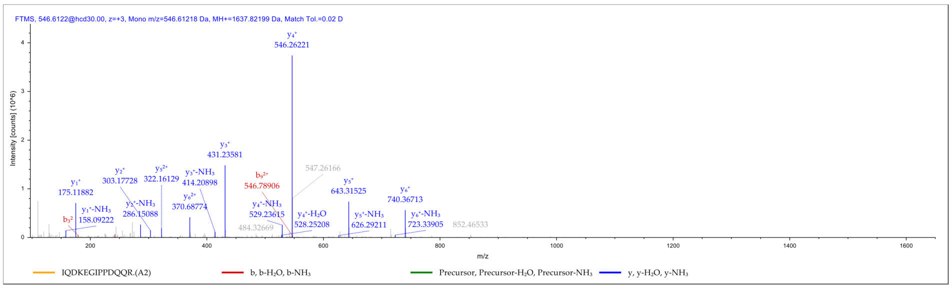

Ubiquitination site: Lys33
Sequence: IQDKEGIPPDQQR, K4-Ub (114.04293 Da)

C

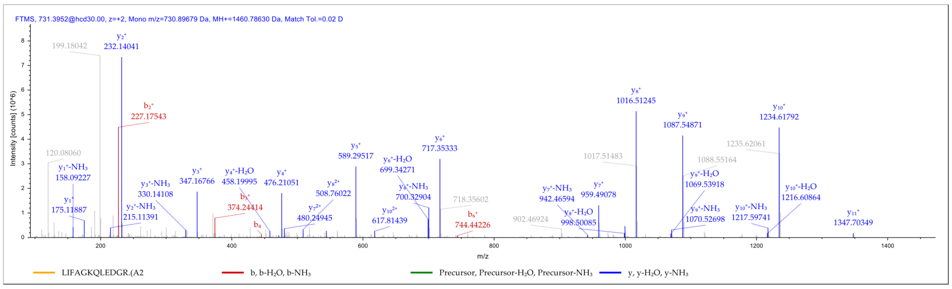

Ubiquitination site: Lys48
Sequence: LIFAGKQLEDGR, K6-Ub (114.04293 Da)

D

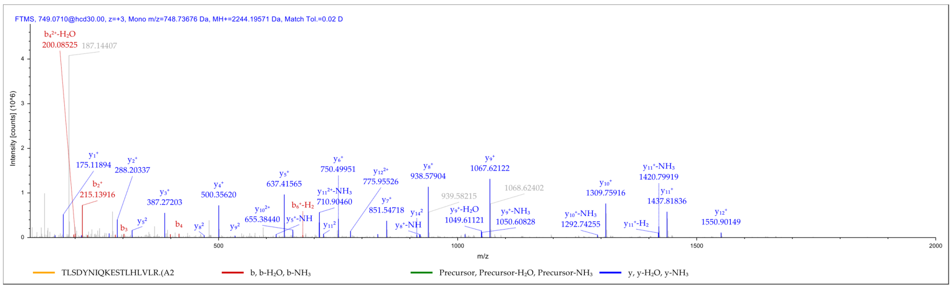

Ubiquitination site: Lys63
Sequence: TLSDYNIQKESTLHLVLR, K9-Ub (114.04293 Da)

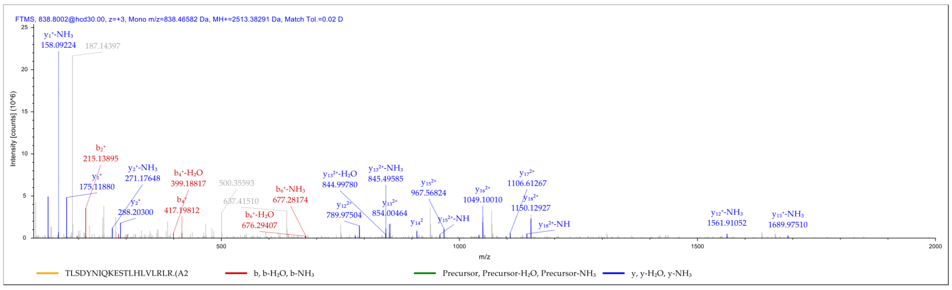

Ubiquitination site: Lys63
Sequence: TLSDYNIQKESTLHLVLRLR, K9-Ub (114.04293 Da)

Figure S15 MS/MS spectra of ubiquitin ubiquitination sites found in the reaction with free KlPCNA and without KlMms2.

A

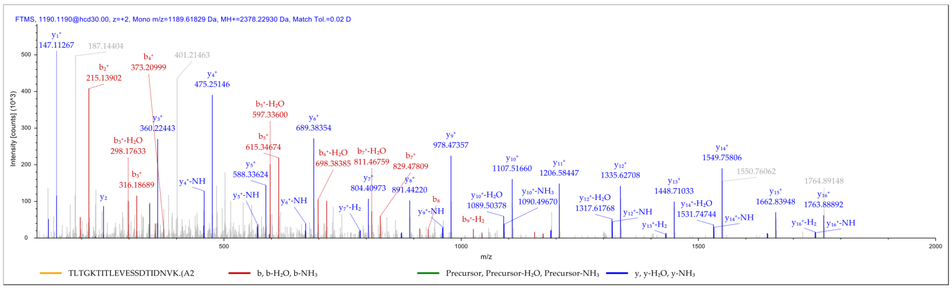

Ubiquitination site: Lys11
Sequence: TLTGKTITLEVESSDTIDNVK, K5-Ub (114.04293 Da)

B

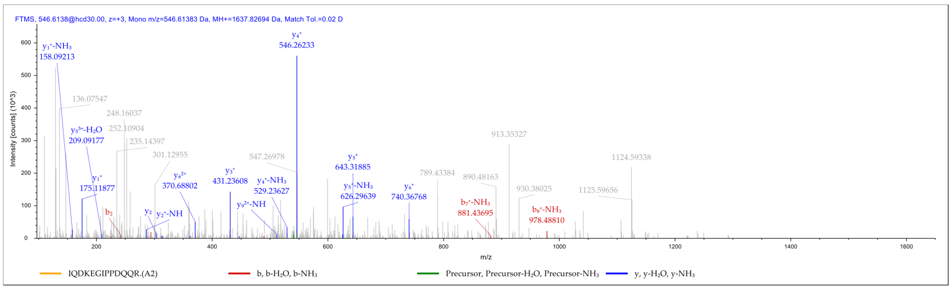

Ubiquitination site: Lys33
Sequence: IQDKEGIPPDQQR, K4-Ub (114.04293 Da)

C

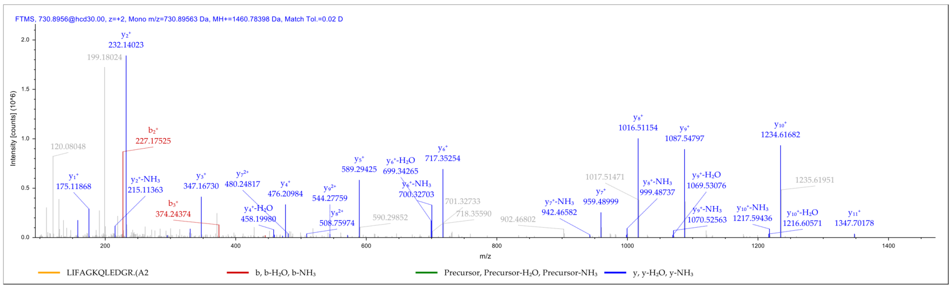

Ubiquitination site: Lys48
Sequence: LIFAGKQLEDGR, K6-Ub (114.04293 Da)

D

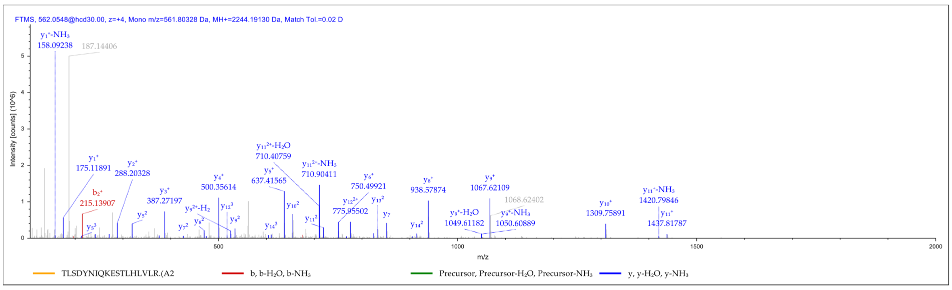

Ubiquitination site: Lys63
Sequence: TLSDYNIQKESTLHLVLR, K9-Ub (114.04293 Da)

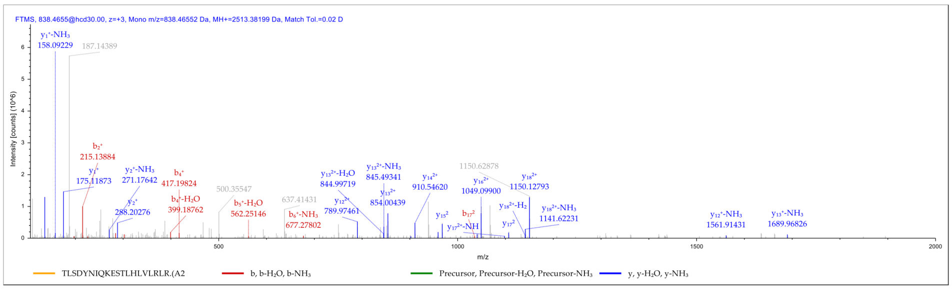

Ubiquitination site: Lys63
Sequence: TLSDYNIQKESTLHLVLRLR, K9-Ub (114.04293 Da)

Figure S16 MS/MS spectra of ubiquitin ubiquitination sites found in the reaction with DNA loaded KlPCNA and without KlMms2.

A

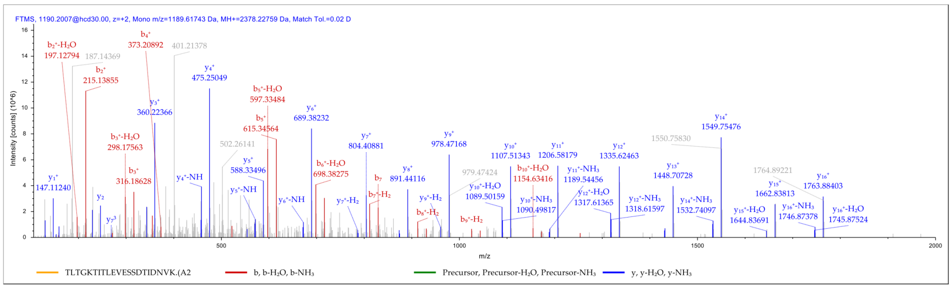

Ubiquitination site: Lys11
Sequence: TLTGKTITLEVESSDTIDNVK, K5-Ub (114.04293 Da)

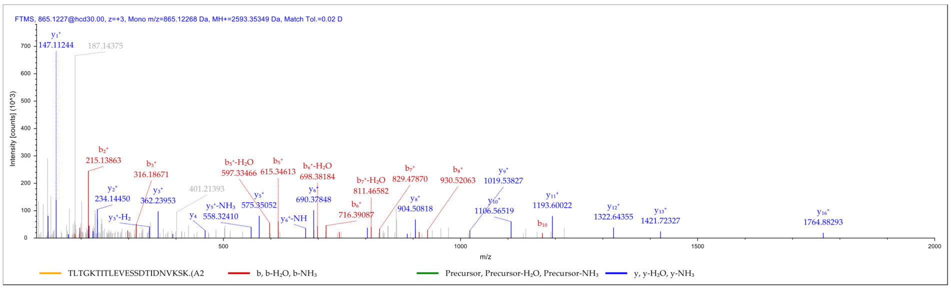

Ubiquitination site: Lys11
Sequence: TLTGKTITLEVESSDTIDNVKSK, K5-Ub (114.04293 Da)

B

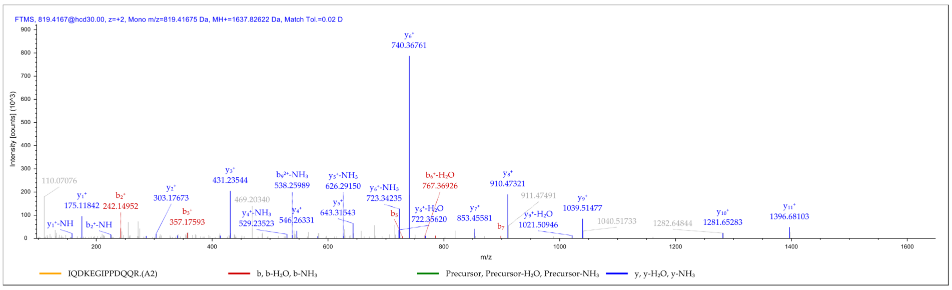

Ubiquitination site: Lys33
Sequence: IQDKEGIPPDQQR, K4-Ub (114.04293 Da)

C

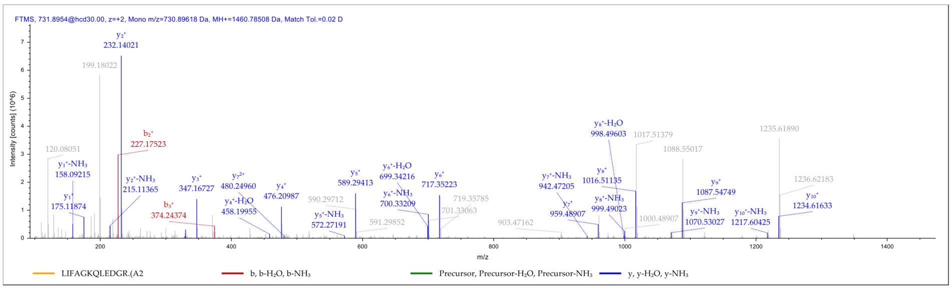

Ubiquitination site: Lys48
Sequence: LIFAGKQLEDGR, K6-Ub (114.04293 Da)

Continues on the next page

Continued from the previous page

D

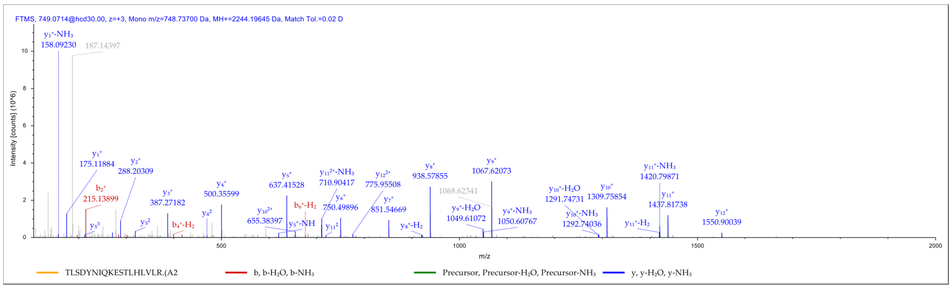

Ubiquitination site: Lys63
Sequence: TLSDYNIQKESTLHLVLR, K9-Ub (114.04293 Da)

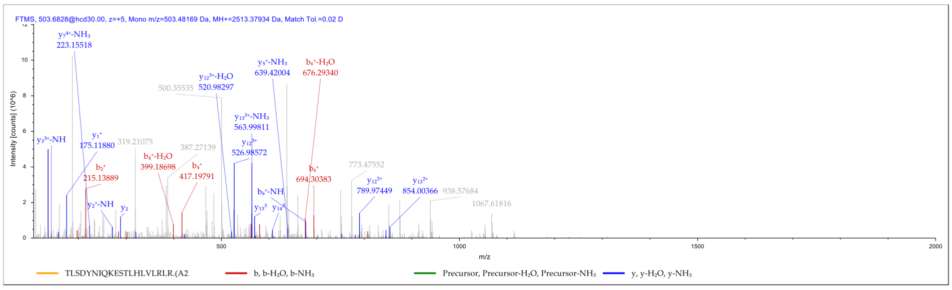

Ubiquitination site: Lys63
Sequence: TLSDYNIQKESTLHLVLRLR, K9-Ub (114.04293 Da)

Figure S17 MS/MS spectra of ubiquitin ubiquitination sites found in the reaction with free KlPCNA and KlMms2.

A

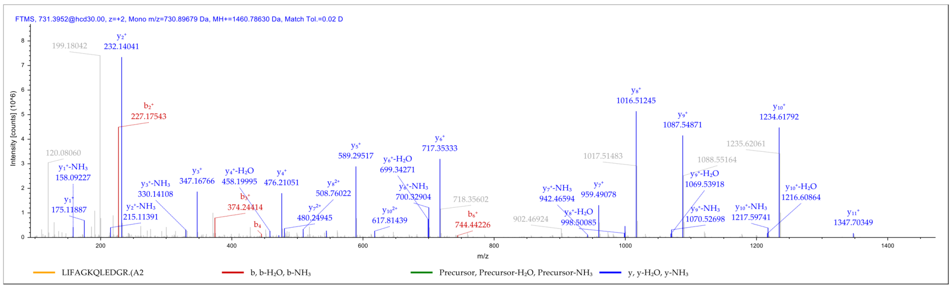

Ubiquitination site: Lys48
Sequence: LIFAGKQLEDGR, K6-Ub (114.04293 Da)

B

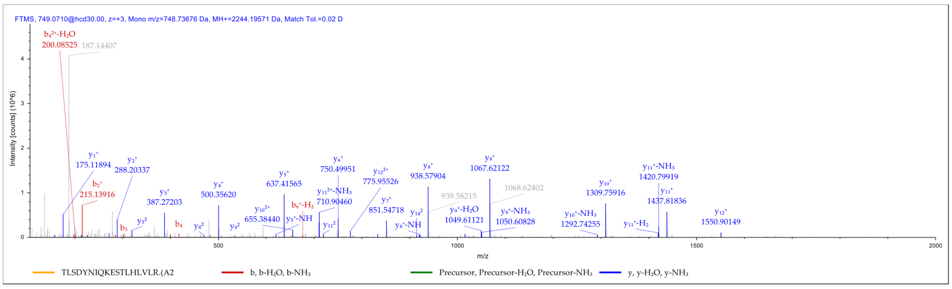

Ubiquitination site: Lys63
Sequence: TLSDYNIQKESTLHLVLR, K9-Ub (114.04293 Da)

Figure S18 MS/MS spectra of ubiquitin ubiquitination sites found in the reaction with DNA loaded KlPCNA and KlMms2.

A

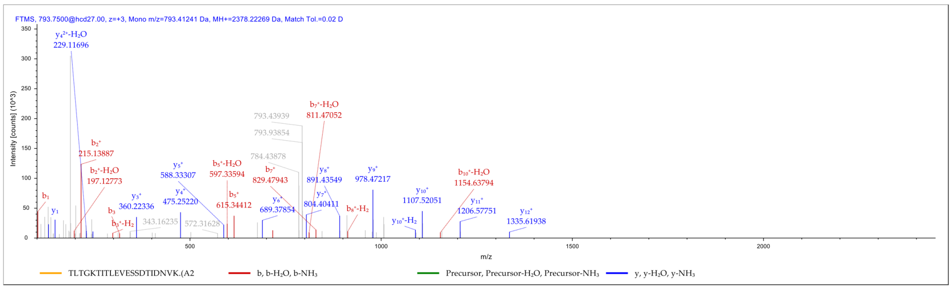

Ubiquitination site: Lys11
Sequence: TLTGKTITLEVESSDTIDNVK, K5-Ub (114.04293 Da)

B

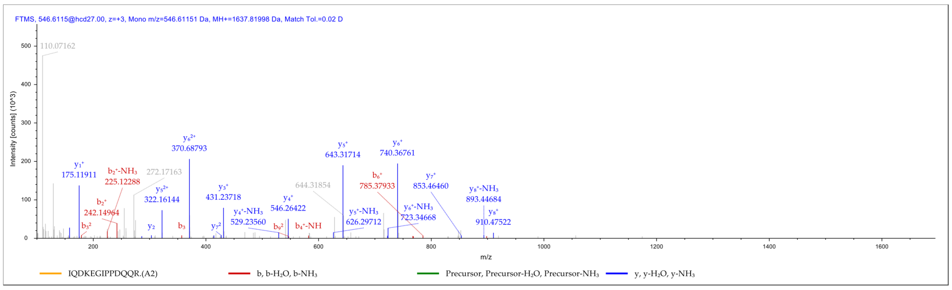

Ubiquitination site: Lys33
Sequence: IQDKEGIPPDQQR, K4-Ub (114.04293 Da)

C

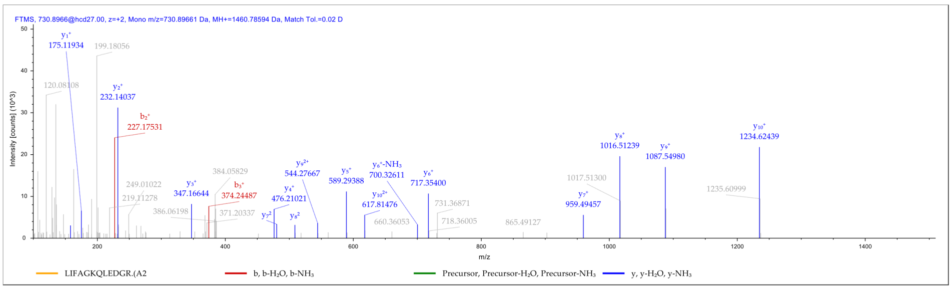

Ubiquitination site: Lys48
Sequence: LIFAGKQLEDGR, K6-Ub (114.04293 Da)

D

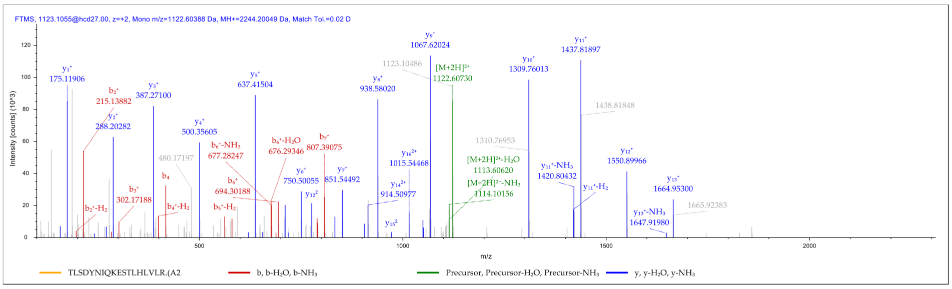

Ubiquitination site: Lys63
Sequence: TLSDYNIQKESTLHLVLR, K9-Ub (114.04293 Da)

Figure S19 MS/MS spectra of ubiquitin ubiquitination sites found in the reaction with free 6KR-substituted KlPCNA and the wild type ubiquitin.

A

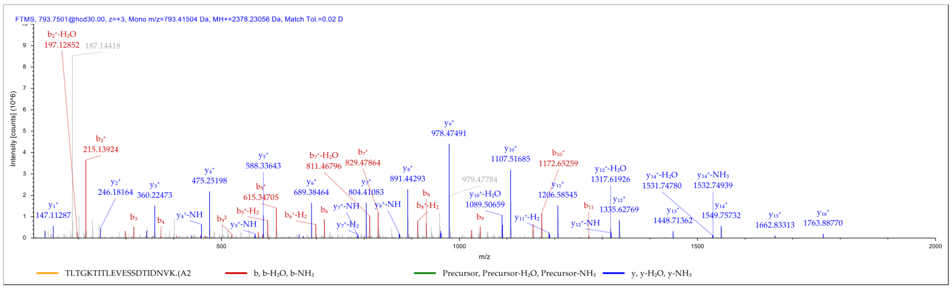

Ubiquitination site: Lys11
Sequence: TLTGKTITLEVESSDTIDNVK, K5-Ub (114.04293 Da)

B

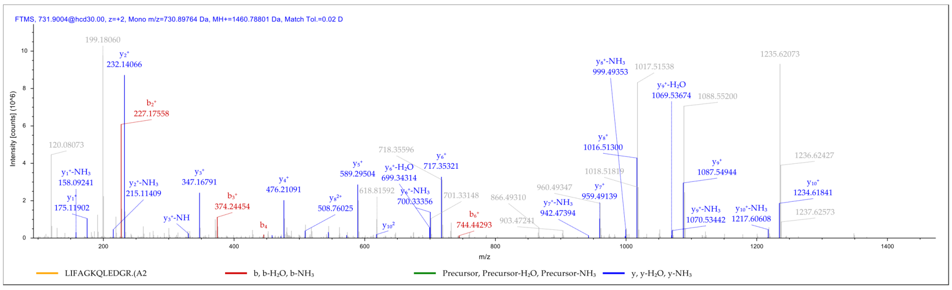

Ubiquitination site: Lys48
Sequence: LIFAGKQLEDGR, K6-Ub (114.04293 Da)

C

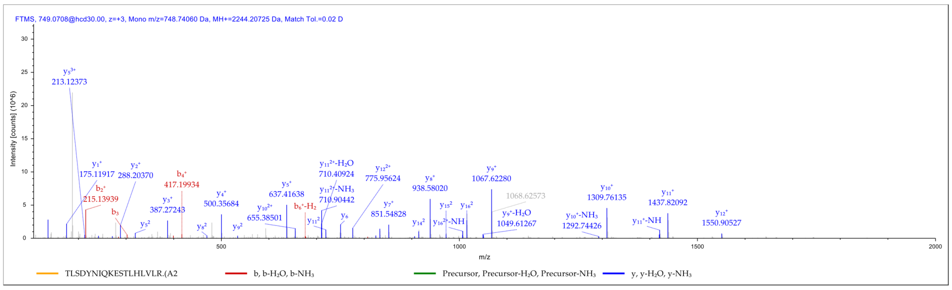

Ubiquitination site: Lys63
Sequence: TLSDYNIQKESTLHLVLR, K9-Ub (114.04293 Da)

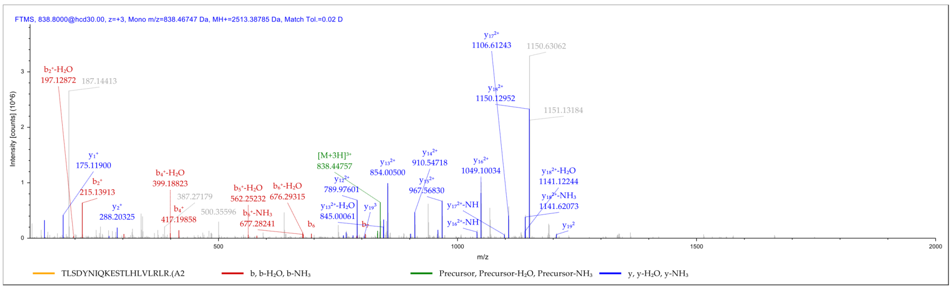

Ubiquitination site: Lys63
Sequence: TLSDYNIQKESTLHLVLRLR, K9-Ub (114.04293 Da)

Figure S20 MS/MS spectra of ubiquitin ubiquitination sites found in the reaction with DNA loaded 6KR-substituted KlPCNA and the wild type ubiquitin.
